# Simeprevir, daclatasvir, and sofosbuvir for hepatitis C virus‐infected patients: Long‐term follow‐up results from the open‐label, Phase II IMPACT study

**DOI:** 10.1002/hsr2.145

**Published:** 2020-02-22

**Authors:** Eric Lawitz, Fred Poordad, Julio A. Gutierrez, Maria Beumont, Greet Beets, Ann Vandevoorde, Pieter Van Remoortere, Donghan Luo, Leen Vijgen, Veerle Van Eygen, Mohamed Gamil

**Affiliations:** ^1^ Texas Liver Institute University of Texas Health Science Center San Antonio Texas; ^2^ Transplant and HPB Institute St. Vincent Medical Center Los Angeles California; ^3^ Janssen Research & Development Janssen Pharmaceutica NV Beerse Belgium; ^4^ Janssen Research & Development LLC Titusville New Jersey

**Keywords:** decompensation, hepatitis C, portal hypertension, simeprevir, sofosbuvir

## Abstract

**Background and aims:**

Direct‐acting antiviral agents (DAAs) for hepatitis C virus (HCV) infection have resulted in high rates of sustained virologic response (SVR) following 8 to 24 weeks of treatment. However, difficult‐to‐cure/cirrhotic patients typically require a longer treatment duration and less is known regarding the long‐term durability of SVR or effect on liver disease progression; to assess this, the IMPACT study followed patients for a 3‐year period after end of treatment.

**Methods:**

The Phase II, open‐label, nonrandomized IMPACT study assessed the efficacy, safety, and pharmacokinetics of the combination of three DAAs (simeprevir, sofosbuvir, and daclatasvir) in HCV genotype 1/4‐infected, treatment‐naïve/‐experienced cirrhotic patients with portal hypertension or decompensated liver disease. Patients from a single site in the United States were assigned to one of two groups by Child–Pugh (CP) score: CP A, CP score less than 7 and evidence of portal hypertension; CP B, CP score of 7 to 9. All patients received simeprevir 150 mg, daclatasvir 60 mg, and sofosbuvir 400 mg once‐daily for 12 weeks between September 2014 and August 2015. All 40 patients included in the study (male, 63%; median age, 58.5 years) achieved SVR 12 and 24 weeks after end of treatment, and the combination was well tolerated.

**Results:**

All patients who reached the 3‐year follow‐up timepoint maintained SVR (CP A, 15/15; CP B, 18/18). CP scores and Model for End‐stage Liver Disease scores remained relatively stable, and mean FibroScan and FibroTest scores declined. No new safety signals were identified.

**Conclusions:**

In the IMPACT study, virologic response to simeprevir, sofosbuvir, and daclatasvir was durable over 3 years (http://ClinicalTrials.gov number: NCT02262728).

**Abbreviations**
AE: adverse event
CP: Child–Pugh
DAA: direct‐acting antiviral agent
EOT: end of treatment
GT: genotype
HCV: hepatitis C virus
ITT: intent‐to‐treat
MELD: Model for End‐stage Liver Disease
QD: once daily
RAV: resistance‐associated variant
SAE: serious adverse event
SVR: sustained virologic response
SVR12: sustained virologic response 12 weeks after end of treatment

## 1 INTRODUCTION

In 2015, it was estimated that 71 million individuals worldwide had chronic hepatitis C virus (HCV) infection.^1^ HCV infection is a leading cause of chronic liver disease,^2^, ^3^ with many patients developing liver cirrhosis or hepatocellular carcinoma.^4^ Furthermore, patients who develop decompensated liver disease have decreased survival rates compared with those patients with compensated cirrhosis.^5^

Current guidelines recommend the use of interferon‐free combinations of direct‐acting antiviral agents (DAAs) for the treatment of HCV infection.^6^, ^7^ Favorable efficacy and tolerability have been demonstrated with these regimens following treatment durations of 8 to 24 weeks (dependent on HCV genotype [GT] and patient characteristics).^6^ However, difficult‐to‐cure patients, including those with cirrhosis, typically require a longer treatment duration.^6^ In addition, the presence of decompensated liver disease may result in impaired hepatic metabolism, affecting the plasma concentrations of the DAAs used.^8^

Simeprevir, sofosbuvir, and daclatasvir are DAAs with non‐overlapping resistance profiles, different mechanisms of action, and different metabolic pathways that target chronic HCV infection.^9^, ^10^ Simeprevir is an HCV NS3/4A protease inhibitor with antiviral activity against GTs 1, 2, 4, 5, and 6^11^, ^12^; sofosbuvir is a pangenotypic nucleotide HCV NS5B polymerase inhibitor^13^; and daclatasvir is a pangenotypic HCV NS5A replication complex inhibitor.^10^, ^14^

The Phase II IMPACT study (http://clinicaltrials.gov number: NCT02262728) was the first to assess the combination of simeprevir, sofosbuvir, and daclatasvir for 12 weeks in HCV GT1‐ or 4‐infected treatment‐naïve or ‐experienced patients with Child–Pugh (CP A) cirrhosis with portal hypertension, or decompensated liver disease (CP B), with a planned 5‐year follow‐up period.^10^ As published previously, all 40 patients (100%) achieved sustained virologic response (SVR)12 and SVR24, and the 3‐DAA combination was well tolerated. During the long‐term follow‐up phase, the study sponsor decided to cease their HCV clinical development program.^15^ Therefore, this manuscript presents the results of the final analysis for the long‐term follow‐up period of the study (reduced to up to 3 years after the end of treatment [EOT]).

## 2 METHODS

The study design, methodology, key inclusion and exclusion criteria, and procedures of this trial have been reported previously.^10^

### 2.1 Patients and study design

In brief, IMPACT was a Phase II, open‐label study carried out at a single site in the United States. The study comprised a screening phase of approximately 4 weeks, a 12‐week open‐label treatment phase, and a posttreatment long‐term follow‐up phase. During the treatment phase, patients received simeprevir 150 mg once daily (QD), daclatasvir 60 mg QD, and sofosbuvir 400 mg QD for 12 weeks. For all patients, a posttreatment follow‐up phase was scheduled for a total period of 5 years, during which patients attended follow‐up visits every 6 months. As mentioned previously, the follow‐up period was subsequently shortened to 3 years.

The study included both treatment‐naïve and interferon‐based (± ribavirin) HCV treatment‐experienced patients of at least 18 years of age with chronic HCV GT1‐ or 4‐infection and cirrhosis (defined as a FibroScan® score >14.5 kPa at screening). Liver disease was classified by CP score; CP A, score less than 7 with documented portal hypertension; CP B, score 7 to 9.

### 2.2 Procedures

During the long‐term follow‐up period of the study, efficacy (as assessed by SVR [HCV RNA less than 15 IU/mL; detectable or undetectable]), safety, change in Model for End‐stage Liver Disease (MELD), CP, FibroScan, and FibroTest (BioPredictive, Paris, France) scores were assessed.

#### 2.2.1 Detection of HCV RNA

Blood samples were collected at predefined time points during the long‐term follow‐up period, at the 1‐, 1.5‐, 2‐, 2.5‐, and 3‐year follow‐up visits, and plasma was subsequently isolated. RNA extraction and quantification of HCV RNA was performed in a central laboratory using the COBAS® AmpliPrep/COBAS® TaqMan® HCV Quantitative Test v2.0 (Roche; lower limit of quantification: 15 IU/mL). Further details of this methodology have been reported previously^10^ (see appendix S1 of the previous IMPACT article).

#### 2.2.2 Assessment of liver disease status

Liver disease status was monitored by the assessment of CP, MELD, FibroTest, and FibroScan scores, which were assessed at screening or baseline and during follow‐up.

#### 2.2.3 Safety

During the long‐term follow‐up phase, reporting of adverse events (AEs) was limited to all serious AEs (SAEs) only.

### 2.3 Objectives

The objectives of the long‐term follow‐up phase were to assess the durability of SVR in the IMPACT study and the effect of treatment on liver disease progression.

### 2.4 Statistics

Since this was an exploratory analysis within an exploratory study, no formal sample size calculation was performed; however, it was considered that a total sample size of 40 patients would be sufficient to explore the primary and secondary objectives, as reported previously.^10^ All efficacy analyses were performed on the intent‐to‐treat (ITT) population (all enrolled patients who had taken at least one dose of any study drug). The endpoints were analyzed overall and by CP class using descriptive statistics, using SAS version 9.4.

### 2.5 Ethics

The study was approved by IntegReview IRB, a regional Institutional Review Board in Austin, Texas, and met the principles of the Declaration of Helsinki. All patients provided written informed consent.

## 3 RESULTS

### 3.1 Patient disposition

**Figure 1:**
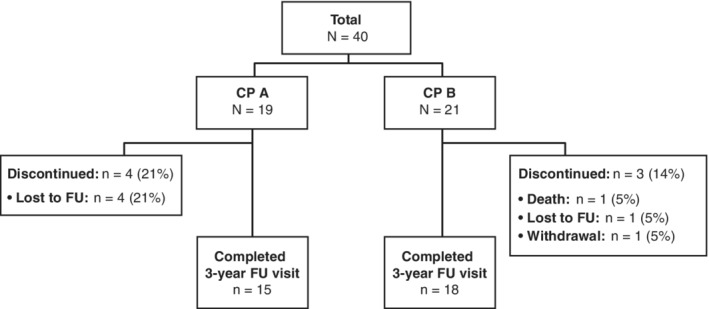
Patient disposition at the 3‐year follow‐up visit. CP, Child–Pugh; FU, follow‐up

In total, 74 patients were screened for the IMPACT study. All of the 40 patients enrolled in the treatment phase (19 patients in the CP A group [patients with documented portal hypertension and CP score <7] and 21 patients in the CP B group [patients with CP score 7‐9]) entered the long‐term follow‐up phase (Figure 1). In the CP A and CP B groups, 79% (15/19) and 86% (18/21) of patients completed their 3‐year follow‐up visit, respectively. Of the remaining patients, five were lost to follow‐up (CP A group, 21% [4/19]; CP B group, 5% [1/21]), one withdrew from the study (CP B group, 5% [1/21]), and one patient died (CP B group, 5% [1/21]). The median (interquartile range [IQR] as Q1; Q3) follow‐up time was 1105.5 (1041.0; 1142.0) days (equivalent to 36.3 [34.2; 37.5] months) during the IMPACT study.

### 3.2 Baseline demographics and disease characteristics

The majority of patients were male (63% [25/40]), with HCV GT1a infection (65% [26/40]), and a median age of 58.5 years. In the CP A group (n = 19), portal hypertension was diagnosed by the presence of upper gastrointestinal (GI) varices in all patients. A mean baseline MELD score of 10.1 was reported for the CP B group (n = 21), and 95% of these patients (20/21) had clinical features of decompensation (ascites, 81% [17/21]; hepatic encephalopathy, 67% [14/21]; median albumin, 3.2 g/dL). A full description of the patient characteristics has been reported previously.^10^

### 3.3 Efficacy

**Figure 2:**
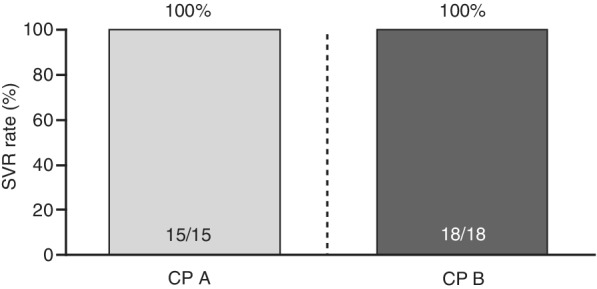
Sustained virologic response at 3‐year follow‐up. CP, Child–Pugh; SVR, sustained virologic response

**Figure 3:**
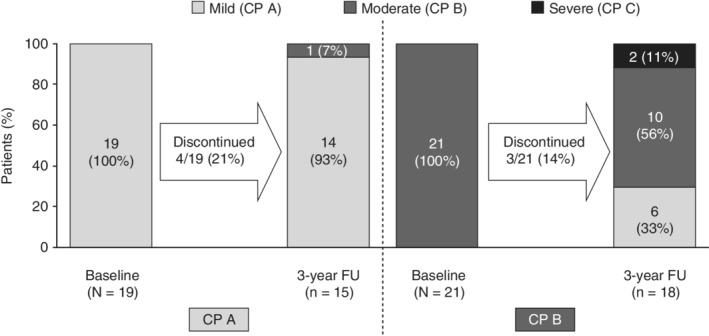
Change in Child–Pugh scores between the baseline visit and the 3‐year follow‐up visit. CP, Child–Pugh; FU, follow‐up

All patients remaining in the study at the 3‐year follow‐up visit (15/15 in the CP A group and 18/18 in the CP B group) had maintained their SVR, and no late viral relapse was observed (Figure 2). Figure 3 shows the change in CP score from baseline to the 3‐year follow‐up visit. In the CP A group, the majority of patients (14/15, 93%) remained stable with mild disease at the 3‐year follow‐up visit, and one patient had an increase in score from CP A (mild disease) to CP B (moderate disease). In the CP B group, 10/18 patients (56%) remained stable with a moderate disease score at the 3‐year follow‐up visit, and there was an improvement in liver disease (decrease in CP score from CP B to CP A) in 6/18 patients (33%). Two of the 18 patients (11%) in the CP B group progressed to severe liver disease (CP C) at the 3‐year follow‐up visit. There were no new reports of hepatic encephalopathy or esophageal varices from the SVR12 time point to the 3‐year follow‐up visit. No patients reported severe/refractory ascites in the CP A group (one had mild‐to‐moderate ascites), and most patients in the CP B group had mild‐to‐moderate ascites during the 3‐year follow‐up visit except for one patient in the CP B group who developed severe/refractory ascites at the 2‐year follow‐up visit, which continued until the 3‐year follow‐up visit.

**Figure 4:**
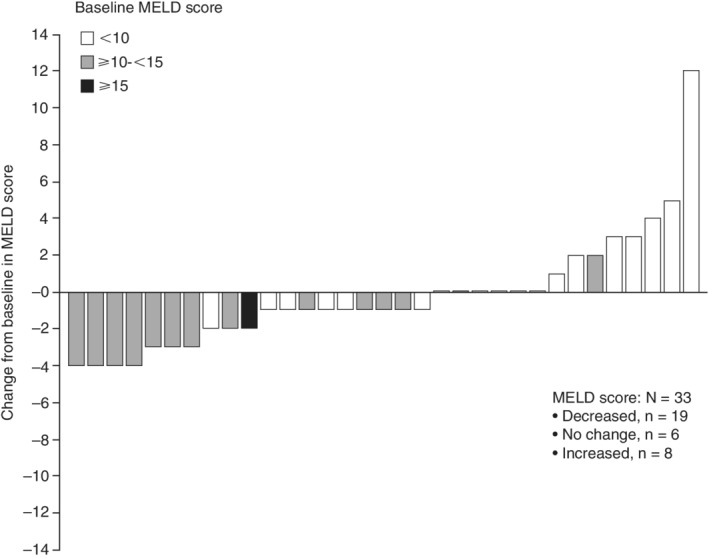
Individual changes from baseline in MELD scores at the 3‐year follow‐up visit, by baseline score. MELD, Model for End‐stage Liver Disease

**Figure 5:**
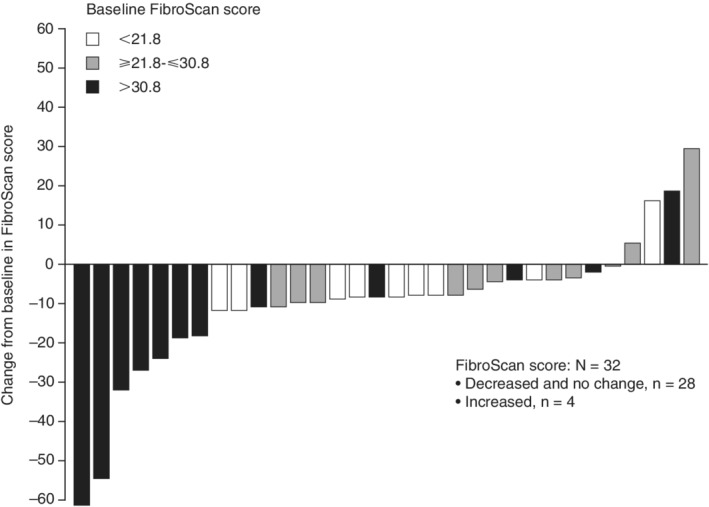
Individual changes from baseline in FibroScan scores at the 3‐year follow‐up visit, by baseline score

Individual MELD scores decreased from baseline to the 3‐year follow‐up visit in 18/32 (56%) patients (Figure 4). The median (IQR) change from baseline in MELD scores remained stable at 3‐year follow‐up: −1 (−1; 0) for patients in the CP A group and −1 (−3; 2) for patients in the CP B group. Individual FibroScan scores decreased or did not change from baseline at 3‐year follow‐up in 88% (28/32) of patients (Figure 5). The median (IQR) change from baseline in FibroScan scores was −8.9 (−12.2; −8.1) for patients in the CP A group and −6.8 (−11.2; −0.7) for patients in the CP B group. The median (IQR) change from baseline in FibroTest scores was −0.11 (−0.22; 0.03) for patients in the CP A group and −0.1 (−0.21; 0.07) for patients in the CP B group.

### 3.4 Safety

During the treatment phase, there were no deaths or AEs that led to treatment discontinuation.^10^ During the 3‐year follow‐up period, 4/19 patients (21%) in the CP A group and 9/21 patients (43%) in the CP B group experienced an SAE. Of these, 4/21 patients (19%) in the CP B group experienced de novo hepatocellular carcinoma (HCC). Other SAEs reported in two patients or more within the CP B group included: abdominal pain (n = 2) and ascites (n = 2). One patient in the CP B group died during the 3‐year follow‐up period (due to an upper GI bleed). However, no SAEs or deaths were considered to be related to study treatment (Table 1).

**Table 1:** Summary of serious adverse events during long‐term (3‐year) follow‐up^a^

Any SAE, n/N (%)	13/40 (32.5%)
SAE related to any study therapy, n/N (%)	0
SAE with fatal outcome, n/N (%)	1/40 (2.5%)
**Preferred term**	**Onset from start of treatment**
*CP A patients (n = 4)* b	
Cerebrovascular accident	Day 427
Hepatic lesion^c^	Day 702
Skin ulcer (worsening)	Day 514
Diverticulitis	Day 832
Gastrointestinal stromal tumor	Day 920
*CP B patients (n = 9)* d	
Hepatic encephalopathy	Day 692
Abdominal pain	Day 573
Abdominal pain	Day 1031
Encephalopathy	Day 1141
Ascites	Day 949
Ascites	Day 1121
De novo hepatocellular carcinoma	Day 949
De novo hepatocellular carcinoma	Day 367
De novo hepatocellular carcinoma	Day 921
De novo hepatocellular carcinoma	Day 1017
GI hemorrhage	Day 685
Death (upper GI bleed)	Day 729

Abbreviations: CP, Child–Pugh; GI, gastrointestinal; SAE, serious adverse event.

a Collected between the Week 24 and 3‐year follow‐up visits.

b One patient had 2 SAEs: skin ulcer (worsening) and gastrointestinal stromal tumor.

c Benign arteriovenous malformation.

d One patient had three SAEs: two incidences of ascites, and de novo hepatocellular carcinoma; and, one patient had two SAEs: abdominal pain and de novo hepatocellular carcinoma.

## 4 DISCUSSION

The Phase II IMPACT study assessed the combination of simeprevir, sofosbuvir, and daclatasvir for 12 weeks in HCV GT1‐ or 4‐infected, treatment‐naïve, or ‐experienced patients with portal hypertension or decompensated liver disease. These three DAAs have different mechanisms of action and metabolic pathways, with non‐overlapping resistance profiles.^9^, ^10^ This was the first clinical study using this combination of treatment to evaluate shorter than 24 weeks of treatment in patients with decompensated liver disease. The 3‐year follow‐up period allowed an investigation into the effects of long‐term DAA treatment on disease progression and the durability of SVR in patients with advanced liver disease.

During the long‐term follow‐up phase of this study, the sponsor decided not to continue their HCV clinical development program. This decision was not driven by a safety concern. Irrespective of this decision, the 3‐year follow‐up data analyzed here, provides insight into durability of response in patients with decompensated liver disease and, therefore, it was considered to be of significant relevance to the field.

The SVR results from the final visit of the IMPACT study, 3 years after EOT, revealed that all patients remaining in the study (15/15 in the CP A group and 18/18 in the CP B group) maintained SVR. Despite the fact that simeprevir is no longer marketed, these results are encouraging because they suggest that combining DAAs with different mechanisms of action can lead to the achievement of long‐term SVR in difficult‐to‐cure patients.

There have been few reports regarding the long‐term durability of SVR to DAA treatment. A recent study by Kozbial et al of 551 patients with advanced fibrosis, decompensated or compensated cirrhosis and SVR after interferon, and ribavirin‐free DAA therapy found that eradication of HCV was durable irrespective of the DAA combination used.^16^ However, the median (range) length of follow‐up in that study was only 65.6 (13.0‐155.3) weeks. In another study, by Hayashi et al, with a mean (range) follow‐up period of 21.5 (4.8‐30.3) months, late relapse in patients who achieved SVR with daclatasvir and asunaprevir was rare (4 of 413 patients) and comparable with that seen following interferon therapy.^17^

The results of the IMPACT study also provide insight into the long‐term effects of such treatment regimens on liver function. The CP scores for the majority of patients in the CP A and CP B groups remained stable; where CP scores differed from baseline at the 3‐year follow‐up visit, more patients' CP scores improved rather than worsened by the 3‐year time point. In both CP A and CP B groups, mean change from baseline in MELD scores remained stable from baseline to the 3‐year time point, while individual and mean FibroScan and mean FibroTest scores generally decreased from baseline in both CP A and CP B groups. Individual FibroScan improvements in liver stiffness were most pronounced in those patients with the highest stiffness scores at baseline. This may be due to the regression of liver fibrosis or reductions in inflammation associated with SVR.^18^ No new safety signals for this combination treatment were identified during long‐term follow‐up in this study and, overall, this DAA regimen was well tolerated. While liver‐related SAEs were rare among patients in the CP A group, several patients in the CP B group experienced liver‐related SAEs, including ascites, hepatic encephalopathy, and de novo HCC. In the CP B group, one patient died due to an upper GI bleed, which was not considered to be related to the study treatment.

In this study, four patients developed de novo HCC following SVR. A recent review article summarizing the results of 11 studies examining the incidence of HCC following DAA treatment reported a de novo incidence rate of 0% to 7.4%, with the authors commenting that their review does not suggest that there is a higher rate of de novo HCC occurrence or recurrence after DAA therapy.^19^ Furthermore, it has also been suggested that SVR is associated with a significant decrease in de novo or recurrent HCC,^20^ dissipating concerns of DAA treatment being associated with subsequent occurrence of HCC. When compared with interferon‐based regimens, evidence has indicated that there may be a mild increased risk of de novo HCC with DAA treatment.^21^ However, interferon‐based regimens can only be given with caution to patients with cirrhosis and only if they have sufficient liver function and minimal portal hypertension.^21^

This was an exploratory study, and as such, there were several limitations, including the open‐label design and that the study was conducted at a single center. There was also a relatively small sample size, which only included patients with CP A and B stage of liver disease. In addition, the posttreatment follow‐up was shortened to 3 years, following the discontinuation of the sponsor's HCV clinical development program. At the time this study was conducted, the MELD score was used as a measure of change in liver function as it was the most suitable measurement for patients with CP A and B. The recently reported Albumin‐Bilirubin grades^22^ for the assessment of liver disease in those with mild deterioration of liver function have since been proven to be a more sensitive marker of liver function in the setting of mild dysfunction and in HCC.

In conclusion, HCV eradication by triple DAA therapy provided durable SVR and a good clinical prognosis in HCV‐infected patients. To further assess the long‐term clinical benefit of achieving SVR in patients with advanced liver disease, studies involving greater patient numbers and longer durations of follow‐up would be required.

## FUNDING INFORMATION

Janssen Research & Development funded this study; contributed to the study design; participated in the collection, analysis, and interpretation of data; and participated in the preparation and approval to submit this manuscript.

## CONFLICT OF INTEREST

E.L. has attended Advisory Committees or Review Panels for AbbVie, Achillion Pharmaceuticals, Bristol‐Myers Squibb, Enanta Pharmaceuticals, Gilead Sciences, Janssen, Merck & Co., Novartis, Regulus, Santaris Pharmaceuticals, and Theravance; has received grant/research support from AbbVie, Achillion Pharmaceuticals, Bristol‐Myers Squibb, Enanta Pharmaceuticals, Gilead Sciences, GlaxoSmithKline, Janssen, Merck & Co., Roche, Salix, Santaris Pharmaceuticals, Tacere, and Theravance; and has performed speaking and teaching for AbbVie, Bristol‐Myers Squibb, Gilead Sciences, and Janssen. F.P. has attended Advisory Committees or Review Panels for Abbott/AbbVie, Achillion Pharmaceuticals, Boehringer Ingelheim, Bristol‐Myers Squibb, Genentech, Gilead Sciences, Idenix, Inhibitex, Janssen, Merck & Co., Novartis, Pfizer, Salix, and Vertex and has received grant/research support from AbbVie, Achillion Pharmaceuticals, Anadys, Boehringer Ingelheim, Bristol‐Myers Squibb, Genentech, Gilead Sciences, Idenix, Merck & Co., Novartis, Pharmasset, Salix, Tibotec/Janssen, and Vertex. J.A.G. has attended Advisory Committees or Review Panels for AbbVie, Bristol‐Myers Squibb, Gilead Sciences, Janssen, and Merck & Co. and has received grant/research support from AbbVie, Bristol‐Myers Squibb, Gilead Sciences, Merck & Co., and Tibotec/Janssen. M.B., G.B., A.V., L.V., and M.G. are employees of Janssen Pharmaceutica NV and shareholders of Johnson & Johnson. V.V.E. is an employee of Janssen Pharmaceutica NV. P.V.R. and D.L. are employees of Janssen Research & Development and shareholders of Johnson & Johnson. With the exception of Janssen Research & Development, for which contributions are detailed in the “Funding” section, all other listed companies had no involvement in the study design; collection, analysis, and interpretation of data; or preparation and approval to submit this manuscript.

## AUTHOR CONTRIBUTIONS

Conceptualization: Maria Beumont, Greet Beets, Ann Vandevoorde, Pieter Van Remoortere, Leen Vijgen, Veerle Van Eygen, and Mohamed Gamil

Formal Analysis: Eric Lawitz, Fred Poordad, Julio A. Gutierrez, Maria Beumont, Greet Beets, Ann Vandevoorde, Pieter Van Remoortere, Donghan Luo, Leen Vijgen, Veerle Van Eygen, and Mohamed Gamil

Investigation: Eric Lawitz, Fred Poordad, Julio A. Gutierrez, Maria Beumont, Greet Beets, Ann Vandevoorde, Pieter Van Remoortere, Leen Vijgen, Veerle Van Eygen, and Mohamed Gamil

Methodology: Maria Beumont, Greet Beets, Ann Vandevoorde, Pieter Van Remoortere, Leen Vijgen, Veerle Van Eygen, and Mohamed Gamil

Writing ‐ Reviewing & Editing: Eric Lawitz, Fred Poordad, Julio A. Gutierrez, Maria Beumont, Greet Beets, Ann Vandevoorde, Pieter Van Remoortere, Donghan Luo, Leen Vijgen, Veerle Van Eygen, and Mohamed Gamil

All authors have read and approved the final version of the manuscript.

Dr Lawitz had full access to all of the data in this study and takes complete responsibility for the integrity of the data and the accuracy of the data analysis.

## TRANSPARENCY STATEMENT

Dr Lawitz affirms that this manuscript is an honest, accurate, and transparent account of the study being reported, that no important aspects of the study have been omitted, and that any discrepancies from the study as planned (and, if relevant, registered) have been explained.

## DATA ACCESSIBILITY STATEMENT

The data sharing policy of Janssen Pharmaceutical Companies of Johnson & Johnson is available at https://www.janssen.com/clinical-trials/transparency. As noted on this site, investigators and physicians can request access to the clinical study report and participant‐level data if these data will be used for scientific research that will advance medical knowledge and public health. Data can be requested immediately following publication, with no end date. Data requests will be evaluated by an independent review panel—the Yale Open Data Access (YODA) Project—and can be submitted at http://yoda.yale.edu.
